# Biocompatible Silver Nanoparticles: Study of the Chemical and Molecular Structure, and the Ability to Interact with Cadmium and Arsenic in Water and Biological Properties

**DOI:** 10.3390/nano11102540

**Published:** 2021-09-28

**Authors:** Federica Bertelà, Martina Marsotto, Cecilia Meneghini, Luca Burratti, Valentin-Adrian Maraloiu, Giovanna Iucci, Iole Venditti, Paolo Prosposito, Veronica D’Ezio, Tiziana Persichini, Chiara Battocchio

**Affiliations:** 1 Department of Sciences, Roma Tre University of Rome, Via della Vasca Navale 79, 00146 Rome, Italy; fed.bertela@stud.uniroma3.it (F.B.); martina.marsotto@uniroma3.it (M.M.); giovanna.iucci@uniroma3.it (G.I.); iole.venditti@uniroma3.it (I.V.); veronica.dezio@uniroma3.it (V.D.); tiziana.persichini@uniroma3.it (T.P.); 2 Department of Chemistry, Sapienza University of Rome, P.le A. Moro 5, 00185 Rome, Italy; meneghini.1716330@studenti.uniroma1.it; 3 Department of Industrial Engineering and INSTM, University of Rome Tor Vergata, Via del Politecnico 1, 00133 Rome, Italy; luca.burratti@uniroma2.it (L.B.); paolo.prosposito@uniroma2.it (P.P.); 4 National Institute of Materials Physics, 405A Atomistilor St., 077125 Magurele, Romania; maraloiu@infim.ro

**Keywords:** biocompatible nanoparticles, silver nanoparticles, heavy metals ions, structural characterization, synchrotron-radiation techniques, toxicological profile

## Abstract

In the field of research for designing and preparing innovative nanostructured systems, these systems are able to reveal the presence of heavy metals in water samples, and can efficiently and selectively interact with them, allowing for future applications in the field of water remediation. We investigated the electronic and molecular structure, as well as the morphology, of silver nanoparticles stabilized by mixed biocompatible ligands (the amino acid L-cysteine and the organic molecule citrate) in the presence of cadmium and arsenic ions. The molecular, electronic, and local structure at the ligands/silver nanoparticles interface was probed by the complementary synchrotron radiation-induced techniques (SR-XPS, NEXAFS and XAS). The optical absorption (in the UV-Vis range) of the nanosystem was investigated in the presence of Cd(II) and As(III) and the observed behavior suggested a selective interaction with cadmium. In addition, the toxicological profile of the innovative nanosystem was assessed in vitro using a human epithelial cell line HEK293T. We analyzed the viability of the cells treated with silver nanoparticles, as well as the activation of antioxidant response.

## 1. Introduction

The spread of a wide range of contaminants in surface water and groundwater has become a critical issue worldwide, due to population growth, the rapid development of industrialization and long-term droughts. Contaminants persisting in wastewater include anthropogenic, natural contaminants and aquatic pathogens. As for anthropogenic pollutants, the main contamination sources are the presence of leather tanning, mining and milling, oil spilling and the chemical industries. Among other contaminants, heavy metal ions such as Cu(II), Co(II), Hg(II), Cd(II), Cr(III), Ni(II), Fe(II), and As(III) are known for their toxicity and negative impact on human health and the environment [1,2,3]. These ions are released into the environment through industrial, domestic, and agricultural activities.

Conventional water treatment methods create a heavy impact on the environment: they are often chemically, energetically and operationally intensive, need considerable financial efforts, engineering expertise and infrastructure, accurate maintenance, all of which precludes their use in much of the world. There is an increasing need for more effective, low-energy, low-cost, sustainable and robust methods to decontaminate waters from the source to the end user. In this context, nanotechnology promises to bring huge improvements in water remediation industry [4,5,6,7,8,9]. Nanoparticles can be used as nano-sorbents, in order to create clusters of contaminants, and their typical Light Scattering Plasmon Resonance (LSPR) can be exploited for detection purposes, allowing for their implementation in small-scale devices [10,11]. However, a critical issue in the use of sorbent nanoparticles is their successive release in water. Using AgNPs in the detection and decontamination of water requires an investigation of their potential toxicity to human health and the definition of their cytotoxic effects, in order to ensure their management and relative use. Among the evidence in the literature, the effects of AgNPs on cells mainly concern modifications of cell morphology, alteration of viability and the generation of oxidative stress [12].

In this work, we investigated the interaction between silver nanoparticles, chemically stabilized with l-cysteine and citrate (AgNPs/L-cys/citr) with As(III) and Cd(II) ions in water. The complexity of these nanomaterials requires the contribution of complementary techniques, in order to obtain a most complete and accurate description of the molecular, electronic structure and the morphology of the nano-aggregates, as well as to characterize the nanoparticles–ion metal interaction in terms of the functional groups involved and local geometry at the metal site. To address this issue, we probed the electronic structure and coordination chemistry of the AgNPs and heavy metal ions by SR-XPS; assessed the stability of the organic ligands L-cysteine and citrate by NEXAFS and IRRAS; investigated the local structure around the silver nanoparticles, with XAS at the S-K edge and Ag-K edge; analyzed the nanostructures morphology by HR-TEM. In addition, to define the toxicological profile of AgNPs, we studied their effects on human kidney epithelial cells (HEK293T) taken as a cellular model. Epithelia, indeed, mediated the first interaction between the human body and silver nanoparticles, eventually dispersed in air, water, and soil. Given that AgNPs were known to induce the generation of reactive oxygen species (ROS), we investigated the activation of their antioxidant responses by evaluating the expression of Nrf2-regulated ARE genes such as catalase, superoxide dismutase, system xc−, and glutamate-cysteine ligase.

## 2. Materials and Methods

### 2.1. AgNPs/L-cys/citr Synthesis and Preliminary Characterization

Silver nitrate (AgNO_3_, 99.9% pure), sodium citrate (Na_3_C_6_H_5_O_7_, citr, 99% pure), L-Cysteine (C_3_H_7_NO_2_S, L-cys, 99.9% pure), and sodium borohydride (NaBH_4_, 99.9% pure) were purchased from Sigma Aldrich and used as received. 

The AgNPs, stabilized with LCys and Cit, were synthetized and characterized as reported in previous work [13,14,15]. In 50 mL of distilled water, 1.47 g of sodium citrate were dissolved (0.01 M); in 25 mL of distilled water 0.006 g of L-cysteine were dissolved (0.002 M); and in 25 mL of distilled water 0.21 g of AgNO_3_ (0.05 M) were dissolved. Then, 25 mL of LCys solution, 10 mL of Cit solution and 2.5 mL of AgNO_3_ solution were mixed in a flask. The mixture was stirred and degassed with Argon for 10 min, then 4 mL of NaBH_4_ solution (0.016 g in 4 mL distilled water) were added. The reaction was stopped after 2 h and the AgNPs were purified by centrifugation (13,000 rpm, 10 min, 3 times with deionized water).

### 2.2. UV-Visible Sensing Tests

Regarding the contamination by heavy metal ions, the tests were conducted using the following salts: NaAsO_2_ and Cd(NO_3_)_2_ (purchased from Sigma Aldrich (Milan, Italy) and used without further purification). For these solutions, deionized water was used (electrical resistivity 18.2 MΩ*cm at room temperature). UV-vis spectra were performed by using single-use UV-PMMA cuvettes with Perkin-Elmer Lambda 750 UV-vis-NIR for sensing test characterization. Protocol of sensing tests: a fixed volume of our AgNPs (1.6 mg/mL) was mixed with a fixed volume of heavy metal ions solution at a specific concentration and, after 5 minutes of interaction, the optical absorption spectra were collected. This protocol described here was successfully tested on silver nanoparticles, stabilized by the hydrophilic ligand sodium 3-mercapto-1-propanesulfonate, whose response to several metal ions at different concentrations in the range of few ppm was tested by UV-vis spectroscopy [16].

### 2.3. Advanced Characterization Methods

Synchrotron radiation (SR)-induced X-ray Photoelectron Spectroscopy (SR-XPS) measurements were performed at the materials science beamline (MSB) at the Elettra synchrotron radiation source (Trieste, Italy). MSB was placed at the left end of the bending magnet 6.1 and was equipped with a plane grating monochromator that provided light in the energy range of 21–1000 eV. The base pressure in the UHV end-station was 2 × 10^−10^ mbar. The end-station was equipped with a SPECS PHOIBOS 150 hemispherical electron analyzer, low-energy electron diffraction optics, a dual-anode Mg/Al X-ray source, an ion gun and a sample manipulator with a K-type thermocouple, attached to the rear side of the sample. For this experiment, we detected photoelectrons emitted by C1s, O1s, S2p, Ag3d, N 1s, Cd3d and As3d core levels at normal emission geometry. A photon energy of 630 eV impinging at 60° was selected for all signals to maximize the intensity of sulfur signals, which were very low due to element dilution; the S2p core level was measured with photon energy = 350 eV. Charging correction of binding energies (BEs) was conducted using the aliphatic C 1s as a reference (BE 285.0 eV) [17]. To fit core level spectra, we subtracted a Shirley background and then used Gaussian peak functions as signals components [18,19].

InfraRed Reflection Absorption Spectroscopy (IRRAS) measurements were performed using a Bruker model Vector 22 spectrophotometer. It consisted of a source, a Michelson interferometer, a sample housing compartment, and a DTGS detector. The instrument interfaced with a computer, which used the OPUS program (Bruker) for data acquisition. The spectrophotometer used a Glocar source, consisting of a silicon carbide (SiC) rod that was heated by the Joule effect through a resistor to a temperature, approximately T = 1500 °C. FT-IR spectra in the 4000–1800 cm^−1^ range were recorded in reflectance mode by means of a Specac P/N 19650 series monolayer/grazing angle accessory, at 70° incidence angle of the impinging radiation, with respect to the normal to the sample surface, on samples prepared by depositing a few drops of aqueous solution on substrates consisting of Au/Si(111) wafers.

Near Edge X-ray Absorption Fine Structure (NEXAFS) spectroscopy was carried out at the BEAR beamline (bending magnet for emission absorption and reflectivity) at the ELETTRA storage ring. BEAR was installed at the left exit of the 8.1 bending magnet exit. The apparatus was based on a bending magnet as a source and beamline optics delivering photons from 5 eV up to about 1600 eV with a selectable degree of ellipticity. The UHV end station was equipped with a movable hemispherical electron analyzer and a set of photodiodes to collect angle-resolved photoemission spectra, optical reflectivity, and fluorescence yield. In these experiments, we used ammeters to measure drain current from the sample. C-K edge spectra were collected at magic incidence angle (54.7°) of the linearly polarized photon beam, with respect to the sample surface. In addition, our C-K edge spectra were further calibrated using the resonance at 288.70 eV, assigned to the C=O 1s−π* transition. The raw spectra were normalized to the incident photon flux by dividing the sample spectrum by the spectrum collected on a freshly sputtered gold surface. Spectra were then normalized by subtracting a straight line that fits the part of the spectrum below the edge and assesses to 1 the value at 320.00 eV.

X-ray Absorption Spectroscopy (XAS) measurements at S-K edge (E_o_(S) = 2472 eV) were carried out at room temperature in fluorescence geometry at the XAFS beamline of the Elettra (Trieste, Italy) synchrotron radiation facility [20]. Sample powders (L-Cysteine and NP samples) were painted on carbon tape or filtered on Millipore membranes obtaining thin homogeneous films to maintain negligible reabsorption effects. Several (up to 10) XAS spectra were measured for each sample at a different position, spectra were compared with each other to verify the sample homogeneity, treated using the standard procedures for XAS data normalization and the extraction of the structural XAFS signal [21], and averaged up in order to improve the signal statistics. Data were collected in the near edge (XANES) region as some chlorine contamination (E_o_(Cl) = 2822 eV) did not allow the measure in the extended (EXAFS) region to be extended. The Ag-L_3_ edge ((E_o_(Ag) = 3350 eV) XANES spectra were measured to check the Ag metallic state.

The XANES region is dense with structural and electronic information, especially for complex systems the comparison of data from reference compounds provides reliable details on the absorber coordination chemistry and valence state [22] allowing the S-containing phases present in our samples to be described. For the sake of comparison, the data from the free available database at the ID-21 ESRF beamline were used [23]. Our experimental S-K edge spectrum measured on L-cysteine was aligned to the same from the ID-21 database in order to properly align the energy scale of reference spectra.

Transmission Electron Microscopy (TEM) investigations were carried out using the atomic resolution analytical JEOL JEM ARM200F microscope, operated at 200 kV. The microscope was equipped with a JEOL JED-2300T unit for EDX analysis. The samples were observed in conventional TEM (CTEM) and high-resolution TEM (HRTEM) at magnifications ranging from 8∙103 to 2∙106. The samples were dispersed in ethanol and then sonicated for 3 min. A droplet of this suspension was then deposited onto a 300-mesh carbon lacey TEM Cu grid and allowed to dry at room temperature.

### 2.4. Biological Studies

#### 2.4.1. Materials for Biological Characterization

Dulbecco’s modified Eagle’s medium (DMEM), fetal bovine serum (FBS), 0.25% Trypsin–EDTA solution, gentamicin solution 50 mg/mL, N-acetyl-L-cysteine (NAC) and MTT assay kit were obtained from Sigma-Aldrich (Milan, Italy).

All chemicals were of analytical or reagent grade and were used without further purification.

The Go Taq 2-Step RT-qPCR System Kit was obtained from Promega; the SsoAdvanced Universal SYBR Green Supermix Kit was obtained from Bio-rad Italia (Milan, Italy).

#### 2.4.2. Cell Cultures

HEK293T human epithelial kidney cells were purchased from American Type Culture Collection (Manassas, VA, USA). The cell line was cultured in DMEM supplemented with 10% fetal bovine serum (FBS), 2 mM L-glutamine, 40 μg/mL gentamicin at 37 °C in a humidified 5% CO_2_ incubator. Confluent monolayers of HEK293T cells were subcultured by conventional trypsinization. For the experiments, 5 × 10^4^ or 4 × 10^5^ cells were seeded in 24-well plates or 35 mm tissue culture dishes, respectively, and grown to up to 80% confluence for 18–24 h before treatments. Working colloidal suspension of AgNPs/L-cys/citr were prepared in culture medium from stock solutions stored at +4 °C. Where indicated, cells were treated with 15–25–50–75–100 μg/mL of AgNPs in the absence or presence of 2 mM of N-acetyl-cysteine. Each procedure was performed under a laminar flow hood (Biohazard) to ensure sterile conditions.

#### 2.4.3. MTT Assay

For the experiments to test cell viability, HEK293T cells were seeded in 24-well plates and allowed to grow for 24h. MTT assay was performed as indicated by the manufacturer’s instructions on HEK293T cells at the end of each incubation period. Briefly, MTT solution (stock solution of 0.5 mg/mL) was added to cell cultures at the final concentration of 10%. After incubation at 37 °C for 4 h, formazan crystals were allowed to dissolve in lysis buffer (4 mM of HCl, 0.1% NP40 (*v/v*) in isopropanol) at 37 °C for further 30 min. The optical density (O.D.) of each sample was measured at 570 nm using a microplate reader (BioTek ELx800 Absorbance Microplate Reader, Winooski, VT, USA).

Evaluation of Living and Dead Cells by Trypan Blue exclusion assay [24] was carried out by visually examination and counting HEK293T cells using a light microscope (Nikon, Eclipse TS100). After the treatments, cells were detached and suspended in PBS containing trypan blue and then examined to calculate the percentage of cells that had clear cytoplasm (viable cells) versus cells that had blue cytoplasm (nonviable cells).

##### Gene Expression Analysis

Total RNA was purified by using TRIzol Reagent (Life technologies Italia-Invitrogen, Monza, Italy) and reverse transcribed into cDNA by GoTaq 2-step RT-qPCR system (Promega Italia Srl, Milan, Italy). cDNA was amplified for the following genes: catalase (CAT), superoxide dismutase (SOD2), system xc− (xCT subunit), and glutamate-cysteine ligase (GCLC). Glyceraldehyde 3-phosphate dehydrogenase (GAPDH) mRNA was examined as the reference cellular transcript. The sequences of primers were reported in Table 1. PCR product quantification was calculated by applying the SYBR-Green method. Reactions were performed in Agilent Aria Mx machine (Agilent technologies) using the following program: 45 cycles of 95 °C for 15 s, 60 °C for 60 s, 72 °C for 20 s. GAPDH mRNA amplification products were present at equivalent levels in all cell lysates. The data were calculated relative to the internal housekeeping gene according to the second derivative test (delta–delta Ct (2^−^ΔΔCT) method). The experiments were repeated three times. For each biological assay, three technical replicates were performed.

##### Statistical Analysis

All data were expressed as the mean ± standard error of the mean (SEM) of three independent experiments. Statistical analysis was performed by one-way ANOVA and subsequently by Bonferroni post-test. Differences were considered statistically significant at *p* ≤ 0.05.

## 3. Results and Discussion

Silver nanoparticles stabilized with L-cysteine and citrate were synthesized by a reduction method as extensively reported in [14,15,16]; the nanoparticles response in presence of Cd(II) and As(III) ions was tested, and the obtained adducts were structurally characterized in detail with X-ray, SR-induced spectroscopies and HR-TEM. In addition, the toxicological profile of the pristine AgNPs/L-cys/citr towards human epithelial cells was investigated.

### 3.1. UV-Visible Assessment of AgNPs/L-cys/citr Responsivity and Selectivity for Heavy Metal Ions

The choice of coating the AgNPS with citrate and L-cysteine allowed not only to obtain monodisperse and highly hydrophilic nanoparticles, but also made the AgNPS capable of interacting with the external environment, having sensitivity and selectivity for some heavy metals in the water [14].

**Figure 1:**
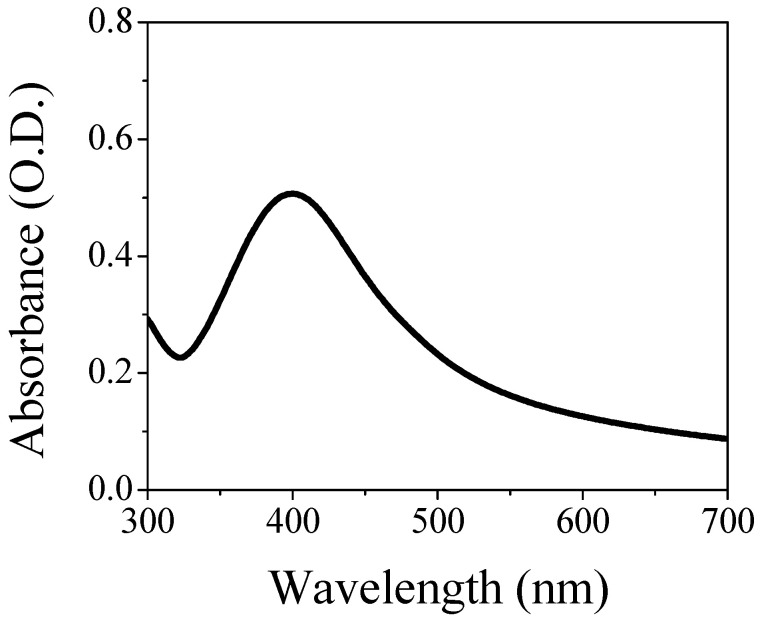
Optical absorption in the UV-Vis range of the AgNPs/L-cys/citr colloidal solution.

Figure 1 shows the LSPR band of the AgNPs/L-cys/citr in a water solution as colloidal dispersion. The energy band has a maximum at 401 nm and a full width at a half maximum (FWHM) equal to 102 nm.

**Figure 2:**
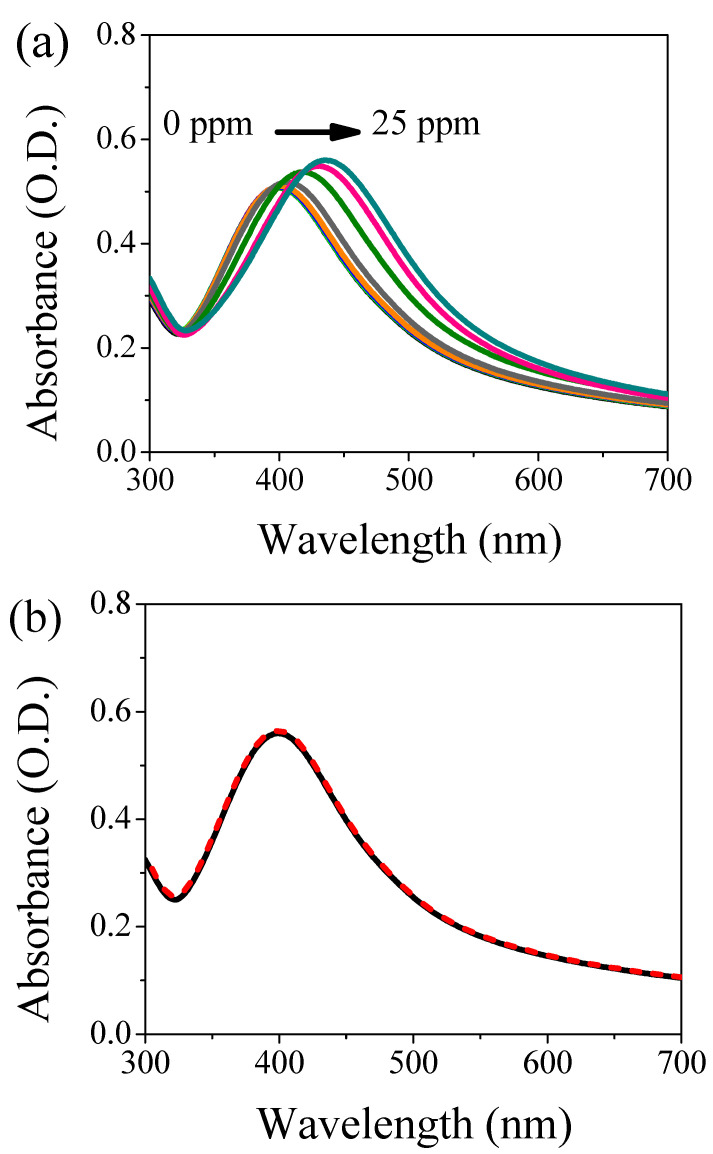
UV-Vis absorption spectroscopy of AgNPs/L-cys/citr in presence of: (**a**) different concentrations of Cd(II) ions (represented with different colors) and (**b**) 25 ppm of As(III) ions where the black curve represents the reference (unpolluted sample), while the dashed red curve refers to the contaminated specimen.

The nanosystem is tested in presence of Cd(II) and As(III). In the case of cadmium cations, the concentration was in the range from 1–25 ppm. Figure 2a shows the UV-Vis absorption spectroscopy of the colloidal solution as a function of Cd(II) content. The peak position shifts toward longer wavelengths (red-shift) increasing the contaminant concentration (for concentration higher than 10 ppm). Moreover, a broadening of the absorption band is also recorded. Figure S1 shows the maximum wavelength of the plasmon band as a function of Cd(II) concentration. Three different regions can be observed: below 5 ppm the AgNPs are not able to detect the presence of cadmium; between 7.5 and 17.5 ppm the optical sensor exhibits a linear behavior, and above 17.5 ppm the system’s saturation occurs. The limit of detection (LOD, 3σ) and the limit of quantification (LOQ, 10σ) can be determined from the linear fit in the linear region. We obtained 9 ppm and 12 ppm as LOD and LOQ, respectively. Although the sensitivity is not very high compared with the literature [25,26,27], and thus the use as an optical sensor for cadmium ions is limited, the interaction between the capping molecules and the contaminating ions is genuine and occurs, even at low concentrations. This prerogative is of fundamental importance; in fact, by exploiting this interaction the system has the potential use as a filter to remove pollutants from the water. A further step is the insertion of our system inside a hydrogel-based matrix for the synthesis of a filter able to remove these toxic ions, improving the applicability and handling in real conditions. The AgNPs/L-cys/citr are also tested in presence of 5, 10 and 25 ppm of As(III), but the LSPR band does not suffer any change in terms of intensity, peak position and band broadening. Figure 2b reports the optical behavior of this nanosystem polluted with 25 ppm of As(III).

### 3.2. Molecular and Electronic Structure: SR-XPS, IRRAS and NEXAFS Studies on AgNPs/L-cys/citr-As(III) and Cd(II)

**Figure 3:**
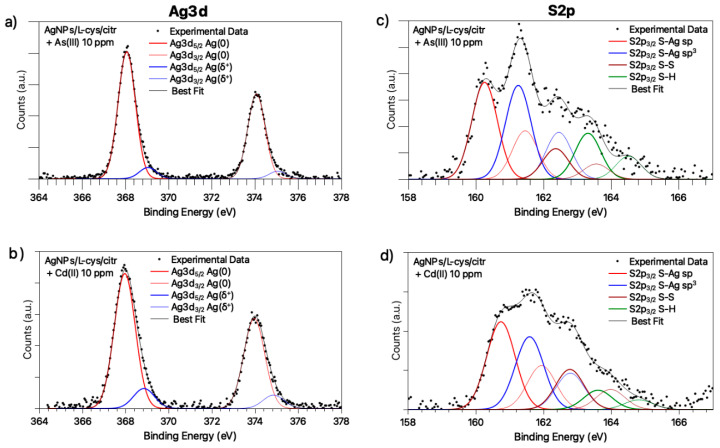
Ag3d core-level spectra of: (**a**) AgNPs/L-cys/citr + As (10 ppm), (**b**) AgNPs/L-cys/citr + Cd (10 ppm); S2p core-level spectra of: (**c**) AgNPs/L-cys/citr + As (10 ppm), (**d**) AgNPs/L-cys/citr + Cd (10 ppm).

Synchrotron radiation-induced X-ray photoelectron spectroscopy (SR-XPS) measurements were applied to investigate the interaction arising between AgNPs/L-cys/citr and heavy metal ions, aiming to understand the different behavior of the plasmon response observed in the presence of Cd(II) or As(III). Experiments were carried out on AgNPs/L-cys/citr exposed to Cd(II) (10 ppm) or As(III) (10 ppm) ions, collecting spectra at C1s, N1s, O1s, Ag3d, S2p, Cd3d and As3d core levels. A table summarizing the main data analysis results (BE (eV), FWHM (eV), relative intensity values and proposed signals assignments) can be found in the Supporting Information file (Table S1). The analysis of C1s, N1s and O1s core levels, in comparison with SR-XPS data analysis already reported for analogous nanomaterials, confirms the stability of silver nanoparticles, and citrate and cysteine capping efficiency [14]: the C1s spectra shows components at 285.00 eV (aliphatic C), 286.5 eV (C-S, C-N), 288.1 eV (C-OH), 289.2 eV and about 291 eV BE (COOH and, respectively, COO^−^); O1s spectra show three different kinds of oxygen atoms, respectively, belonging to the carbonyl (C=O) functional groups (532.0 eV BE), hydroxyl (-OH) moieties (533.0 eV BE) and physisorbed water (small contribution at about 534.5 eV BE). The N1s spectra have a couple of components at about 400 and 401 eV BE, as expected for amine-like nitrogens (in unprotonated and protonated forms, respectively). The C1s, O1s and N1s spectra are reported in Figure S2 in the Supporting Information file. S2p and Ag3d signals are the most indicative for the analysis of the surface–structure of metal nanoparticles capped with thiols; in addition, since -SH moieties are observed to contribute to the ROS reduction in biologic tests, the ability to individuate different S-species is extremely interesting for the evaluation of the toxicological profile of AgNPs stabilized by thiols. Therefore, Ag3d and S2p spectra are reported in Figure 3, and the assignment of both signals spectral components is discussed here in detail; in addition, data analysis results for Ag3d and S2p spectra are summarized in Table 2.

The Ag3d spectra (Figure 3a,b) are asymmetric at a high BE, a common feature in capped nanoparticles [28], indicating that at least two different kinds of silver atoms comprise the nanoparticle: the signal at lower BE values (Ag3d_5/2_ = 368.08 eV) is assigned to metallic silver atoms of the nanoparticle core, while the low-intensity (about 10% of the whole signal) spin-orbit pair at a higher BE (nearly 369 eV) is caused by positively charged silver atoms at the NP surface, interacting with the thiol end-group of L-cysteine [14]. The observed Ag(δ+) atomic *%* are in excellent agreement with the silver nanoparticles’ mean sizes, as suggested by TEM and as will be discussed in the following. Actually, the observed ~10% of silver atoms at the NPs surface suggests a high surface-to-volume ratio, as expected for nanoparticles with a mean diameter close to 5 nm [29].

The S2p spectra, reported in Figure 3c,d, are also composite, showing several spin-orbit pairs indicative for different sulfur-containing chemical groups; the two signal components at a lower BE (S2p3/2 = 161.0 eV; S2p3/2 = 162.0 eV) are indicative of S-Ag bonds with sp and sp^3^-hybridized sulfur atoms, respectively [30]. The spin-orbit pair with the S2p_3/2_ component at about 163.3 eV BE is indicative of R-SH physisorbed thiol moieties. In AgNPs/L-cys/citr + As (10 ppm) and AgNPs/L-cys/citr + Cd (10 ppm) spectra, a feature compatible with disulfide S-S groups is also observed.

**Figure 4:**
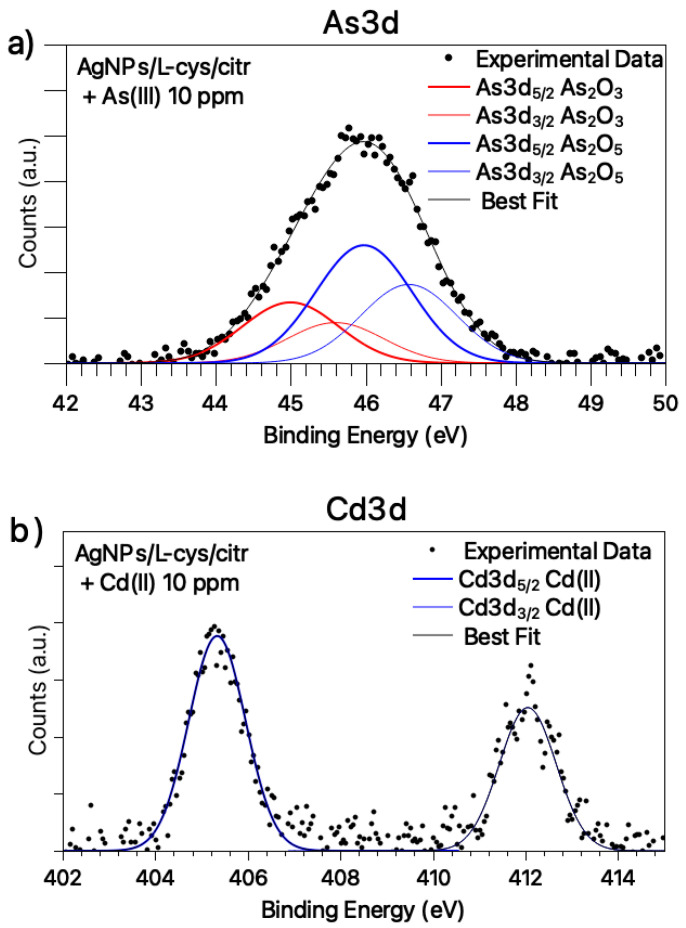
SR-XPS spectra collected at (**a**) Cd3d core-level for silver nanoparticles exposed to Cd(II) solution 10 ppm; (**b**) As3d core-level for silver nanoparticles exposed to As(III) solution 10 ppm.

The Cd3d and As3d core level spectra are also acquired and analyzed. The Cd3d signal (Figure 4a) has a single spin-orbit pair attributed to Cd(II) ions in coordination compounds (BE Cd3d_5/2_ = 405.5 eV) [31,32,33], suggesting that the plasmonic shift observed for AgNPs in the presence of Cd ions could be related to the ability of Cd^2+^ to coordinate the silver nanoparticles by interacting with the carboxylic functional groups of cysteine and/or citrate ligands, as already observed for other bivalent heavy metal ions (Co(II), Ni(II), Hg(II)) and extensively discussed in the literature [16,28]). Conversely, As3d SR-XPS spectrum (Figure 4b) shows two pairs of spin-orbit components indicative for As(III) in As_2_O_3_ (As3d_5/2_ BE = 45.0 eV) [31,34] and As(V) in As_2_O_5_ (As3d_5/2_ BE = 46.9 eV) [31,35], in atomic ratio As(V)/As(III) = 1,9/1; the As(III) partial oxidation to As(V) in water is not surprising, as reported in the literature [36]; however, the presence of arsenic oxides could explain the inability of As ions to coordinate the silver nanoparticles, and, consequently, the absence of a plasmonic response to the presence of this heavy metal ion.

**Figure 5:**
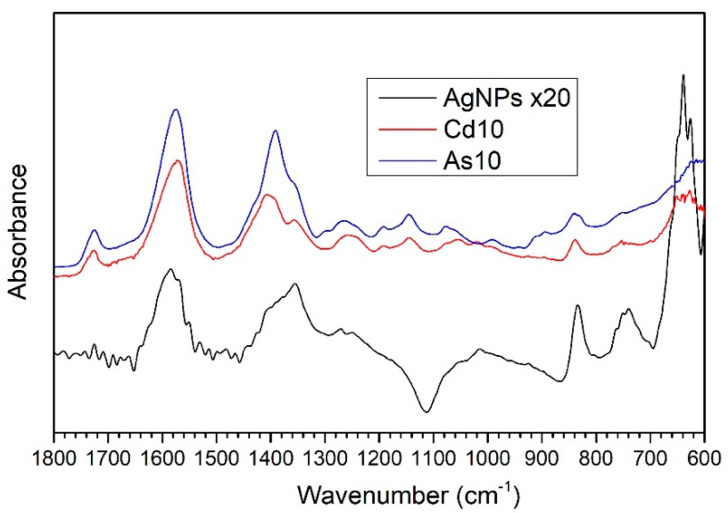
FTIR spectra in the 1800-600 cm^−1^ range of samples As10 and Cd10, treated with 10 ppm solutions of As (III) and Cd (II) ions, respectively, and for the untreated reference sample, AgNPs. The AgNPs reference spectrum was multiplied ×20 in order to obtain a similar intensity for the three spectra.

IRRAS measurements in the 4000–600 cm^−1^ range were performed on samples of AgNPs/L-cys/citr previously suspended in different aqueous solutions containing As(III) and Cd(II) ions at 10 ppm concentration. In addition, measurements were performed on untreated nanoparticles. These measurements allowed us to study the composition and chemical structure of the examined silver nanoparticles. The FTIR spectra in the 1800–600 cm^−1^ range (the region where the most diagnostic bands are located) are shown in Figure 5: AgNPs (black spectrum) represents the reference spectrum related to untreated AgNPs/L-cys/citr; As10 (blue spectrum) corresponds to the AgNPs/L-cys/citr treated with the 10 ppm As(III) aqueous solution; and Cd10 (red spectrum) corresponds to AgNPs/L-cys/citr treated with the 10 ppm Cd(II) aqueous solution. FTIR data showed the higher intensity of Cd10 and, especially, As10 spectra compared to the AgNPs. To illustrate the three spectra on comparable scales we multiplied the AgNPs spectrum ×20 units. For this reason, we consider the As10 and Cd10 spectra as more reliable. Indeed, they present a better signal-to-noise ratio and a lower contamination influence and spurious peaks. These differences are most likely due to non-reproducible characteristics related to sample preparation. All spectra show similarities, especially when comparing the Cd10 and As10 spectra, particularly in the region between 1800 and 600 cm^−1^. The assignment of the main peaks present in the 4000–600 cm^−1^ range of the IR spectra is shown in Table S2 in the Supporting Information [37,38].

The peaks centered at 3360–3340 cm^−1^ (not shown in Figure 5) can be attributed to the O-H-stretching vibrations of the hydroxyl group, coming from the citrate anion and probably from water adsorbed onto nanoparticles surface. The peaks located at 1724 and 1580 cm^−1^ are related to the C=O stretching vibrations of the carboxyl functional group in a protonated (1724 cm^−1^) or deprotonated form (1580 cm^−1^, the asymmetrical stretching of the COO^−^ anion). The main contributions to the symmetric and asymmetric stretching vibrations of the carboxylate group are due to the citrate and L-cysteine molecules, which are present on the AgNPs surface; in fact, the functionalization with Cit and L-cys can also be supported by the presence in the AgNPs spectrum of the peak centered at 1390 cm^−1^, indicating the COO^−^ symmetric stretching vibrations.

Other peaks related to skeletal vibrations of the citrate molecule are listed in Table S2.

Finally, the peak centered at 640 cm^−1^ could be partially due to C-S bonding, though the high intensity of the signal for the AgNPs spectrum suggests a possible superimposition of the C-S peak with C-H bending vibrations, most likely due to surface contamination.

**Figure 6:**
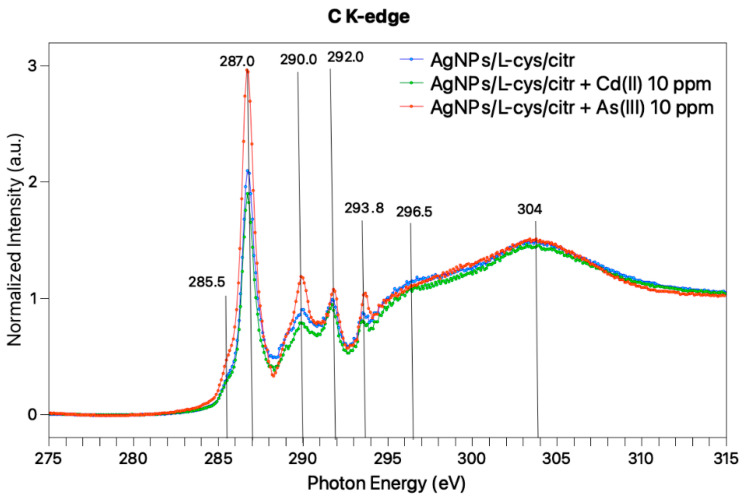
NEXAFS spectra collected at magic angle (57.4°) of AgNPs/L-cys/citr + As (10 ppm) (**orange**) AgNPs/L-cys/citr + Cd (10 ppm) (**green**) and pristine AgNPs/L-cys/citr (**blue**).

Near Edge X-ray Absorption Fine Structure (NEXAFS) spectra were also acquired at the C-K edge for AgNPs treated with As(III) 10 ppm, Cd(II) 10 ppm and, for comparison, pristine AgNPs/L-cys/citr; the three spectra, reported in Figure 6, show features in the same position, confirming that the functional groups of citrate and cysteine are preserved in the AgNPs-heavy metals interaction, as also confirmed by SR-XPS analysis.

At a low photon energy, a sharp resonance located at 287 eV, with a shoulder at 285.5 eV, can be observed for all samples. According to the literature, the 1s-π* transition related to the C=O bond in the carboxylate of L-cys, is expected at 288.6 eV [39], and similar values are expected for carboxyl groups [40]. Moreover, L-cys is expected to show a 1s-σ* peak related to the C-S bond at about 287.3 eV. Therefore, we can assign this band to overlap between π* C=O and σ* C–S transitions. The broad bands observed at higher photon energy values (290, 292, 294, 296 and 304 eV) are indicative for C1s-σ* transitions of C–H, C-N, C-OH, and C=O bonds [31]. On the whole, the three spectra are analogous, confirming the stability of the capping molecules as suggested by IRRAS studies.

### 3.3. Local Structure around Silver Nanoparticles: XAS Measurements at the S-K Edge

**Figure 7:**
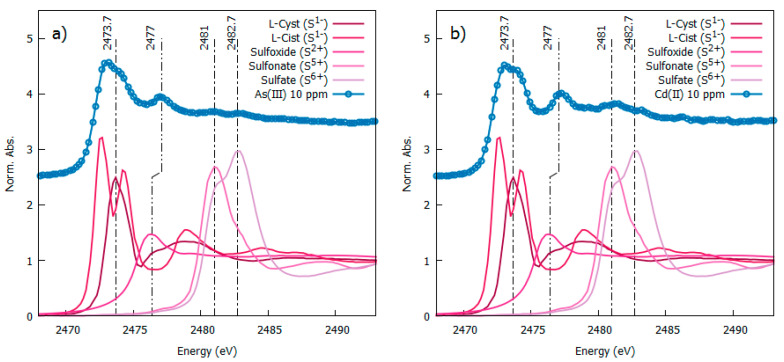
S-K edged spectra measured from 10 ppm As(III) left panel (**a**) and Cd(II)-right panel (**b**) loaded AgNP (vertically shifted fr clarity) in comparison with S-K-edge XANES spectra from reference sulphur compounds [23].

Figure 7 reports the S-K-edge XANES spectra of some sulfur reference compounds and our heavy-metal-loaded AgNPs. The XANES spectra of the reference compounds depict ample shift as a function of the S oxidation state which allows the S oxidation state in our NP to be recognized. The S-K edge measured on AgNPs loaded with As(III) (Figure 7a) and Cd(II) (Figure 7b) presents a white line centered at 2473.7 eV that coincides with the position of the S-K-edge XANES in L-cysteine, pointing out the sulfur, mainly in an S^1-^ valence state. Noticeably the S white line in our heavy-metal-loaded NP is weaker and broader than in L-cysteine. Observing carefully (Figure 7a,b) the white line depicts a double shoulder feature which is largely different from the double peak observed in L-Cystine reference XANES and can be considered the fingerprint of Ag-S coordination, in agreement with the literature [41] pointing out the formation of an Ag-sulfide layer at the NP surface [42]. Based on the spectra of S-reference compounds, the further weak peaks at higher energies can be assigned to some small fraction of oxidized sulfur in the form of sulfoxide (S^2+^ around 2477 eV), sulfonate (S^5+^ around 2481 eV) and sulfate (S^6+^ at 2483 eV), likely due to sample ageing in an aqueous solution. Neither metallic sulfur nor As_2_S_3_ are present in our samples.

The Ag-L_3_ edge spectra measured on As(III) 10 ppm and Cd(II) 10 ppm samples confirm the metallic nature of the Ag (see Figure S3 in the Supplementary Materials).

### 3.4. Silver Nanoparticles Morphology Assessment: TEM

**Figure 8:**
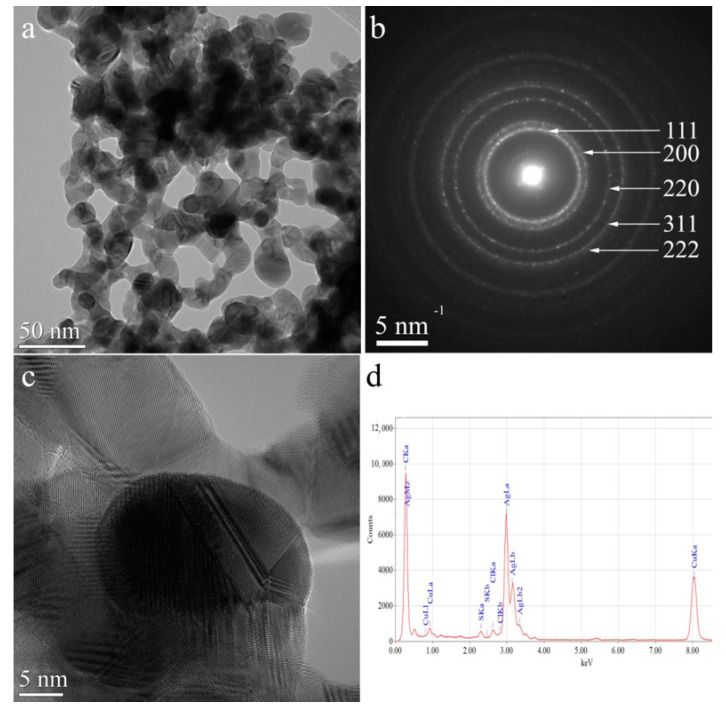
(**a**) CTEM image of AgNPs/L-cys/citr; (**b**) SAED pattern; (**c**) HRTEM image; (**d**) EDS spectrum.

The AgNPs/L-cys/citr morphology was investigated by TEM techniques. CTEM image which is acquired on AgNPs/L-cys/citr is reported in Figure 8a, showing that silver nanoparticles are spherical or highly branched; the diameter of spherical AgNPs ranges from 2 nm up to 15 nm, while branched AgNPs lobs have diameters between 9 nm to 28 nm. The HRTEM image shows that the nanoparticles are well crystallized (Figure 8c). Selected Area Electron Diffraction (SAED) pattern (Figure 8b) demonstrates that the nanoparticles are crystallized in the cubic structure with the space group FM-3mand the lattice parameter a = 4.07724 Å. Energy-Dispersive X-Ray Spectroscopy (EDS) reveals the presence of S and Cl in small concentrations beside Ag (sample), C and Cu (TEM grid).

### 3.5. Biological Characterization

#### 3.5.1. Effect of AgNPs/L-cys/Citron Cell Viability

**Figure 9:**
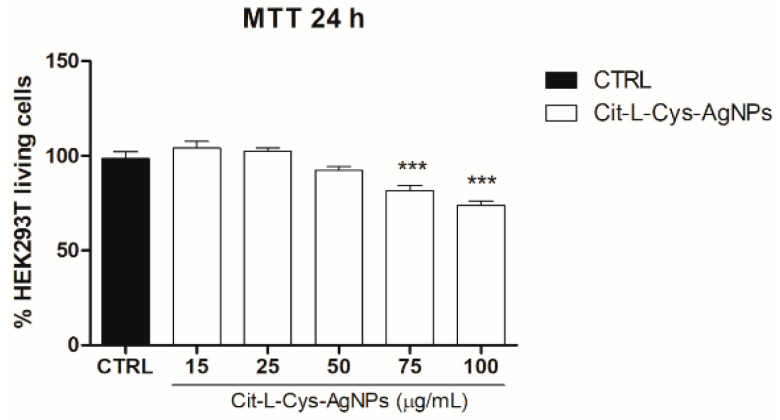
Effect of AgNPs/L-cys/citr on the viability of HEK293T cells. MTT assay was performed on cells treated with cit-L-cys-AgNPs (15-25-50-75-100 μg/mL). *** *p* < 0.001 vs. CTRL.

In order to investigate the effect of AgNPs/L-cys/citr on the viability of epithelial cells, we performed the MTT assay on HEK293T cells treated with different concentrations of AgNPs/L-cys/citr (15, 25, 50, 75, 100 μg/mL) for 24 h. The results shown in Figure 9 indicated that cyt-L-cys-AgNPs induced a significant reduction in cell viability of about 20% and 25%, at concentrations of 75 and 100 μg/mL, respectively. At lower concentrations, cit-L-cys-AgNPs did not affect cell viability.

#### 3.5.2. Effect of the Antioxidant N-Acetyl-Cysteine on AgNPs/L-cys/citr-Induced Cytotoxicity

**Figure 10:**
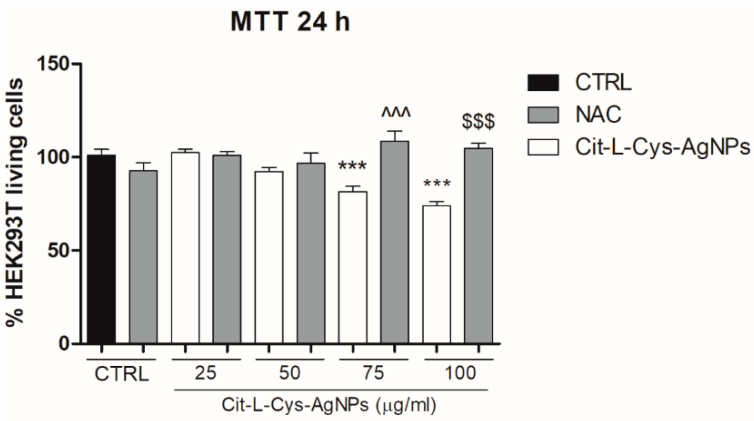
Effect of AgNPs/L-cys/citr on cells viability in presence of NAC. MTT assay on HEK293T pretreated with NAC (2 mM) and treated with cit-L-cys-AgNPs (25-50-75-100 μg/mL) for 24 h. *** *p* < 0.001 vs. CTRL; ^^^ *p* < 0.001 vs. Cit-L-cys-AgNPs 75 μg/mL; $$$ *p* < 0.001 vs. Cit-L-cys-AgNPs 100 μg/mL.

To investigate whether oxidative stress was involved in cell death induced by the higher concentrations of AgNPs/L-cys/citr, we evaluated the cell viability in the presence of N-acetylcysteine (NAC), a ROS scavenger. To this aim, we performed the MTT assay on HEK293T cells pretreated with NAC (2 mM) and treated with AgNPs/L-cys/citr (25, 50, 75, 100 μg/mL) for 24 h. As shown in Figure 10, the results indicated that NAC totally restored the viability of the HEK293T cells at the control level and suggested the involvement of ROS generation in mediating the cytotoxic effect of AgNPs/L-cys/citr on epithelial cells.

#### 3.5.3. Quantification of Dead and Living Cells after AgNPs/L-cys/citr Treatment

To confirm the cytotoxic effect of AgNPs/L-cys/citr and calculate the number of dead cells with respect to the living cells, we performed the trypan blue exclusion assay.

To this end, the HEK293T cells were treated with AgNPs/L-cys/citr (25, 50, 75, 100 μg/mL) for 24 h. The effect of AgNPs/L-cys/citr at a concentration of 100 μg/mL was analyzed in the absence or presence of NAC (2 mM).

**Figure 11:**
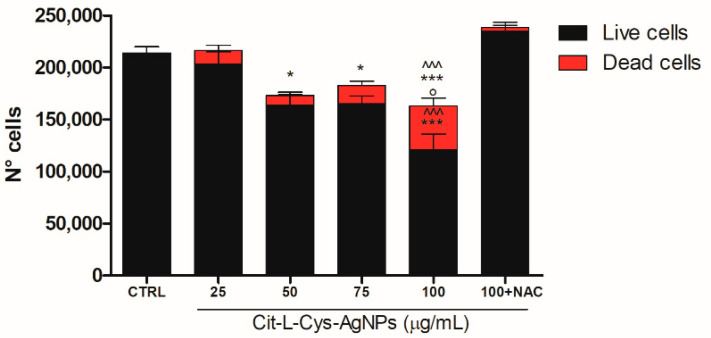
Effect of AgNPs/L-cys/citr on HEK293T cells counting. Number of live and dead cells after 24h from treatments with cit-L-cys-AgNPs (25-50-75-100 μg/mL) and AgNPs/L-cys/citr (100 μg/mL) + NAC (2 mM). Live cells: * *p* < 0.05 vs. CTRL; *** *p* < 0.001 vs. CTRL; ^^^ *p* < 0.001 vs. 100 + NAC; ° *p* < 0.05 vs. 75 μg/mL and 50 μg/mL. Dead cells: *** *p* < 0.001 vs. CTRL; ^^^ *p* < 0.001 vs. 100 + NAC.

As shown in Figure 11, silver nanoparticles were able to significantly reduce the number of living cells already at the 50 μg/mL concentration. At the highest dose of AgNPs/L-cys/citr (100 μg/mL), we observed a significant 3-fold increase in cell death with respect to that induced by 50 μg/mL concentration. As expected, NAC pretreatment was able to completely restore the cell viability of HEK293T treated with the highest dose (100 μg/mL).

Altogether, these results demonstrate that AgNPs/L-cys/citr are able to alter the cell viability of epithelial cells through the induction of oxidative stress.

#### 3.5.4. Effect of AgNPs/L-cys/citr on the Cellular Antioxidant Response

Given that several cell types can activate a specific response to counteract ROS ac-cumulation and oxidative stress, we analyzed the activation of the antioxidant response in epithelial cells treated with AgNPs/L-cys/citr.

In particular, we evaluated the expression of some critical ARE genes regulated by the Nrf2 transcription factor which played a crucial role in antioxidant defense activation. This response was required to maintain the intracellular GSH content and the levels of the antioxidant and cytoprotective enzymes.

To this end, the HEK293T cells were treated with AgNPs/L-cys/citr (100 μg/mL) for 4, 8 and 24 h. At the end of the treatment, the mRNA levels of the ARE genes (GCLC, xCT, CAT and SOD2) were analyzed by Real Time qPCR.

**Figure 12:**
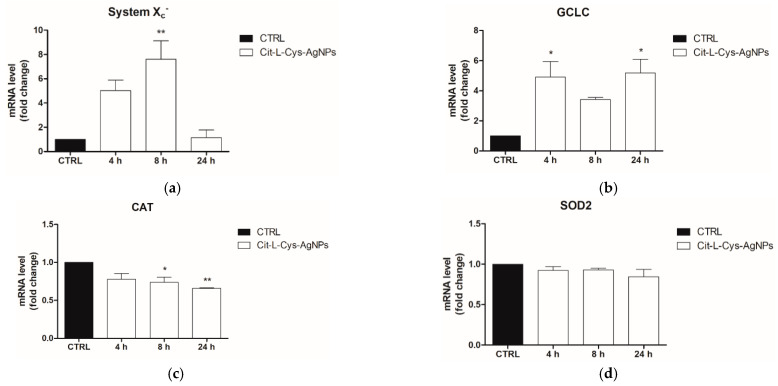
Evaluation of ARE gene expression (mRNA levels) following treatment of HEK293T with AgNPs/L-cys/citr (100mM) by RT-qPCR. Gene analyzed are: (**a**) xCT; (**b**) GCLC; (**c**) SOD2; (**d**) CAT. * *p* < 0.05 vs. CTRL; ** *p* < 0.01 vs. CTRL.

As shown in the graphs of Figure 12, the results demonstrated a significant increase in transcription levels of GCLC, the catalytic subunit of glutamate-cysteine ligase (5-fold) and xCT, the catalytic subunit of System Xc- antiporter, (7-fold) in cells treated with silver nanoparticles, with respect to untreated cells.

In contrast, the mRNA levels of SOD2 remain unchanged with respect to the control, and those of CAT decrease by about 25% and 35% at 8 and 24 h post-treatment, respectively.

As a whole, these data demonstrated a partial activation of the antioxidant response, leading to the increase in mRNA expression only of xCT and GCLC genes, those involved in GSH homeostasis, which was the main antioxidant of the cell. However, the genes coding for catalase and SOD2, enzymes involved in ROS elimination, were not upregulated. Therefore, this partial antioxidant response was not sufficient to counteract the cytotoxic effect elicited by AgNPs/L-cys/citr at the higher concentrations in HEK293T epithelial cells.

## 4. Conclusions

In this work, we investigated the molecular, electronic and chemical structure of silver nanoparticles chemically stabilized with L-cysteine and citrate (AgNPs/L-cys/citr) and exposed to Cd(II) and As(III) ions in an aqueous solution. Due to the high complexity of the proposed nanomaterials, we applied a multi-technique approach to the structural investigation: the electronic structure and coordination chemistry of the AgNPs and heavy metal ions were assessed by SR-XPS, the stability of the organic ligands L-cysteine and citrate was investigated by NEXAFS and IRRAS, and the local structure around the silver nanoparticles was investigated with XAS at the S-K edge and Ag-K edge. In addition, the AgNPs/L-cys/citr morphology was assessed by HR-TEM, showing spherical particles of well-crystallized silver.

Finally, we define the toxicological profiles of AgNPs/L-cys/citr by studying their effects on human kidney epithelial cells (HEK293T) taken as a cellular model. Our findings provide evidence that the nanoparticles did not interfere with epithelial cell viability at concentrations as high as 25 μg/mL, indicating a threshold that must not be exceeded to avoid cytotoxic effects. We also investigated the cellular pathways triggered by AgNPs/L-cys/citr exposition. We analyzed the ability of HEK293T cells to counteract ROS accumulation through the activation of the Nrf2-mediated antioxidant response. The obtained results indicate a cell response which is not completely effective, as suggested by the lack of up-regulation of SOD and CAT genes.

## Figures and Tables

**Table 1:** Sequences of primers used for real-time quantitative PCR.

Gene	Accession Number	PCR Product Size	Nucleotide Sequence
CAT	NM_001752	139 bp	Forward 5′-TCA GGT TTC TTT CTT GTT CAG-3′ Reverse 5′-CTG GTC AGT CTT ATA ATG GAA TT-3′
SOD2	NM_000636.4	199 bp	Forward 5′-AAT GGT GGT GGT CAT ATC A-3′ Reverse 5′-CCC GTT CCT TAT TGA AAC C-3′
System Xc^−^	NM_014331.4	107 bp	Forward 5′-GGT GGT GTG TTT GCT GTC-3′ Reverse 5′-GCT GGT AGA GGA GTG TGC-3′
GCLC	AB262176.1	125 bp	Forward 5′-TTG CAA AGG TGG CAA TGC-3′ Reverse 5′-GAA ACA CAC CTT CCT TCC-3′
GAPDH	NM_002046.7	110 bp	Forward 5′- TTG TTG CCA TCA ATG ACC C -3′ Reverse 5′- CTT CCC GTT CTC AGC CTT G -3′

**Table 2:** BE (eV), FWHM (eV), Atomic percentage and Proposed Assignment for Ag3d and S2p spectral components.

Sample	Signal	BE (eV)	FWHM (eV)	Atomic %	Assignment
AgNPs/L-cys/citr + As (10 ppm)	Ag3d_5/2_	368.09 368.51	0.95 0.95	91.8% 8.2%	Ag (0) Ag+
	S2p_3/2_	160.25 161.25 162.36 163.31	0.89 0.89 0.89 0.89	36.2% 35.0% 11.5% 17.3%	RS-Ag (sp) RS-Ag (sp^3^) RS-SR RS-H physisorbed
AgNPs/L-cys/citr + Cd (10 ppm)	Ag3d_5/2_ Ag3d_5/2_	367.96 368.85	1.14 1.14	87.0% 13.0%	Ag (0) Ag+
	S2p_3/2_	160.74 161.59 162.78 163.61	0.98 0.98 0.98 0.98	39.9% 33.1% 18.2% 8.8%	RS-Ag (sp) RS-Ag (sp^3^) RS-SR RS-H physisorbed

## Supplementary Materials

The following are available online at https://www.mdpi.com/article/10.3390/nano11102540/s1, Figure S1. The maximum wavelength of the absorption band of AgNPs as a function of Cd(II) concentration in ppm. The red line represents the linear behavior in the range from 7.5 to 17.5 ppm. Figure S2: XPS spectra collected at C1s, O1s and N1s core levels; Figure S3: Ag-L3 edge spectra measured on As(III) 10 ppm and Cd(II) 10 ppm samples. Table S1: XPS data for AgNPs/L-cys/citr exposed to Cd(II) and As(III): BE (eV), FWHM (eV) and proposed signals assignments, Table S2: Peak position in wavenumbers (cm−1) and related peak assignment for samples As10 and Cd10, treated with 10 ppm solutions of As(III) and Cd(II) ions, respectively, and for the reference untreated sample AgNPs.
