# Supplementary material for: Biocompatible Silver Nanoparticles: Study of the Chemical and Molecular Structure, and the Ability to Interact with Cadmium and Arsenic in Water and Biological Properties
Source: Nanomaterials (Basel). 2021 Sep 28;11(10):2540. doi: 10.3390/nano11102540 (PMC8541502; doi:10.3390/nano11102540)
Supplement: Supplementary file 1 [file nanomaterials-11-02540-s001.zip › nanomaterials-1390813-supplementary.pdf]

Supplementary

Biocompatible Silver Nanoparticles: Study of the Chemical and Molecular Structure, and the Ability to Interact with Cadmium and Arsenic in Water and Biological Properties

Federica Bertelà [1], Martina Marsotto [1], Cecilia Meneghini [2], Luca Burratti [3], Valentin-Adrian Maraloiu [4], Giovanna Iucci [1], Iole Venditti [1], Paolo Prosposito [3], Veronica D'Ezio [1], Tiziana Persichini [1] and Chiara Battocchio [1,*]

[1] Department of Sciences, Roma Tre University of Rome, Via della Vasca Navale 79, 00146 Rome, Italy; fed.bertela@stud.uniroma3.it (F.B.); martina.marsotto@uniroma3.it (M.M.); giovanna.iucci@uniroma3.it (G.I.); iole.venditti@uniroma3.it (I.V.); veronica.dezio@uniroma3.it (V.D.); tiziana.persichini@uniroma3.it (T.P)
[2] Department of Chemistry, Sapienza University of Rome, P.le A. Moro 5, 00185 Rome, Italy; meneghini.1716330@studenti.uniroma1.it
[3] Department of Industrial Engineering and INSTM, University of Rome Tor Vergata, via del Politecnico 1, 00133, Rome, Italy; luca.burratti@uniroma2.it (L.B.); paolo.prosposito@uniroma2.it (P.P.)
[4] National Institute of Materials Physics, 405A Atomistilor St., 077125 Magurele, Romania; maraloiu@infim.ro
* Correspondence: chiara.battocchio@uniroma3.it

Table S1. XPS data for AgNPs/L-cys/citr exposed to Cd(II) and As(III): BE (eV), FWHM (eV) and proposed signals assignments.

Sample	Signal	BE (eV)	FWHM (eV)	Assignment
AgNPs/L-cys/citr / As10	C1s	285.00	1.36	C-C
		286.55	1.36	C-S; C-N
		288.16	1.36	C-OH
		289.22	1.36	COOH
		290.81	1.36	COO^-
	N1s	400.00	1.78	R-NH_2
		401.35	1.78	R-NH_3^+
	O1s	531.49	1.82	C=O
		532.58	1.82	C-O
		534.38	1.82	H_2O
	$S2p_{3/2}$	160.23	0.83	-S-Ag (sp)
		161.43	0.83	-S-Ag (sp^3)
		163.21	0.83	-SH physisorbed
		162.22	0.83	S-S
		168.24	0.83	oxidized S
	$Ag3d_{5/2}$	368.09	0.95	Ag (0)
		368.51	0.95	Ag^+
	$As3d_{5/2}$	45.00	1.50	As^{+3}
		45.98	1.50	As^{+5}
AgNPs/L-cys/citr / Cd 10	C1s	285.00	1.35	C-C
		286.48	1.35	C-S; C-N
		288.25	1.35	C-OH
		289.60	1.35	COOH
	N1s	399.47	1.22	R-NH_2
		400.59	1.22	R-NH_3^+
	O1s	531.49	1.81	C=O
		532.50	1.81	C-O
		533.95	1.81	H_2O
	$S2p_{3/2}$	160.99	1.26	S-Ag (sp)
		161.90	1.26	S-Ag (sp^3)
		163.36	1.26	SH physisorbed
		168.46	1.26	oxidized S
	$Ag3d_{5/2}$	367.95	1.05	Ag (0)
		368.74	1.05	Ag^+
	$Cd3d_{5/2}$	405.33	1.44	Cd^{2+}

Table S2. Peak position in wavenumbers (cm^{-1}) and related peak assignment for samples As10 and Cd10, treated with 10 ppm solutions of As(III) and Cd(II) ions, respectively, and for the reference untreated sample AgNPs. The peaks are labelled as strong (s), medium (m), weak (w) or shoulder (sh).

Wavenumber (cm^{-1})	Assignment	As10	Cd10	AgNPs
3360-3340	O-H stretching	s	s	
2940-2920; 2850	C-H stretching	w	w	w
1724	C=O stretching carboxylic acid	s	s	
1575-1580	carboxylate CO_2^- asymmetric stretching	s	s	s
1390	carboxylate CO_2^- asymmetric stretching	s	s	sh

1352	C-H bending	sh	sh	s
1260	Citrate skeletal vibration	s	s	
1190	Citrate skeletal vibration	w	w	
1146	Citrate skeletal vibration	w	w	
1074	Citrate skeletal vibration	w	w	
835	CO_2^- bending	w	w	m
750-740		w	w	m
640	C-S stretching	w	m	s

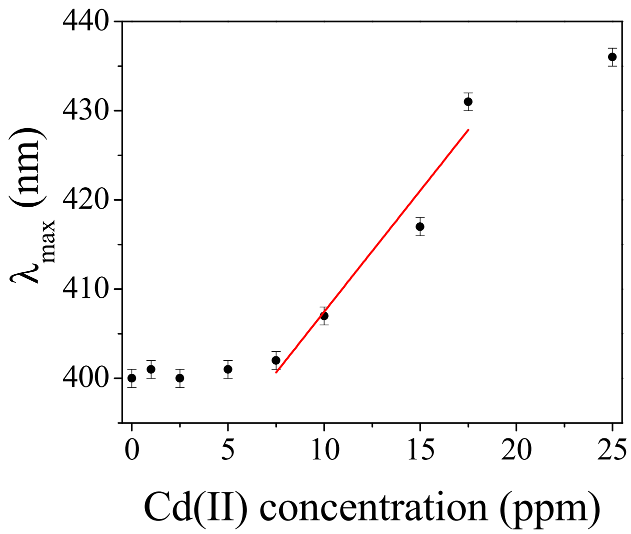

Figure S1. The maximum of the absorption band of AgNPs as a function of Cd(II) concentration in ppm. The red line represents a linear fit in the range from 7.5 to 17.5 ppm.

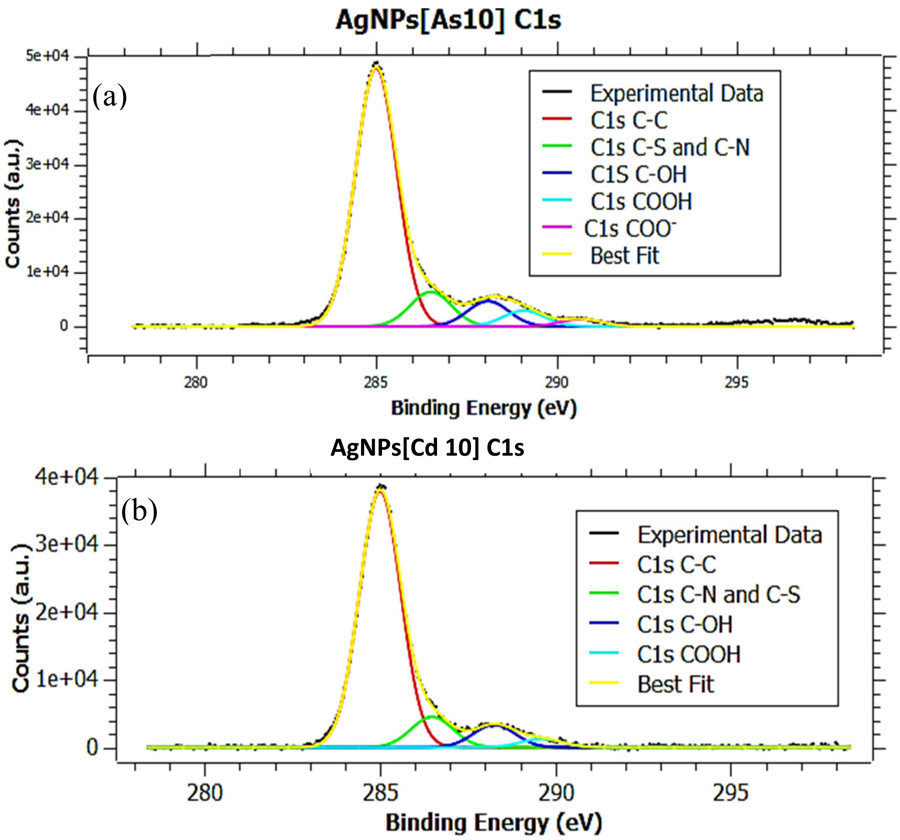
AgNPs[As10] C1s
(a)
Experimental Data
C1s C-C
C1s C-S and C-N
C1S C-OH
C1s COOH
C1s COO-
Best Fit
Counts (a.u.)
Binding Energy (eV)
5e+04
4e+04
3e+04
2e+04
1e+04
0
280
285
290
295
AgNPs[Cd 10] C1s
(b)
Experimental Data
C1s C-C
C1s C-N and C-S
C1s C-OH
C1s COOH
Best Fit
Counts (a.u.)
Binding Energy (eV)
4e+04
3e+04
2e+04
1e+04
0
280
285
290
295

C1s

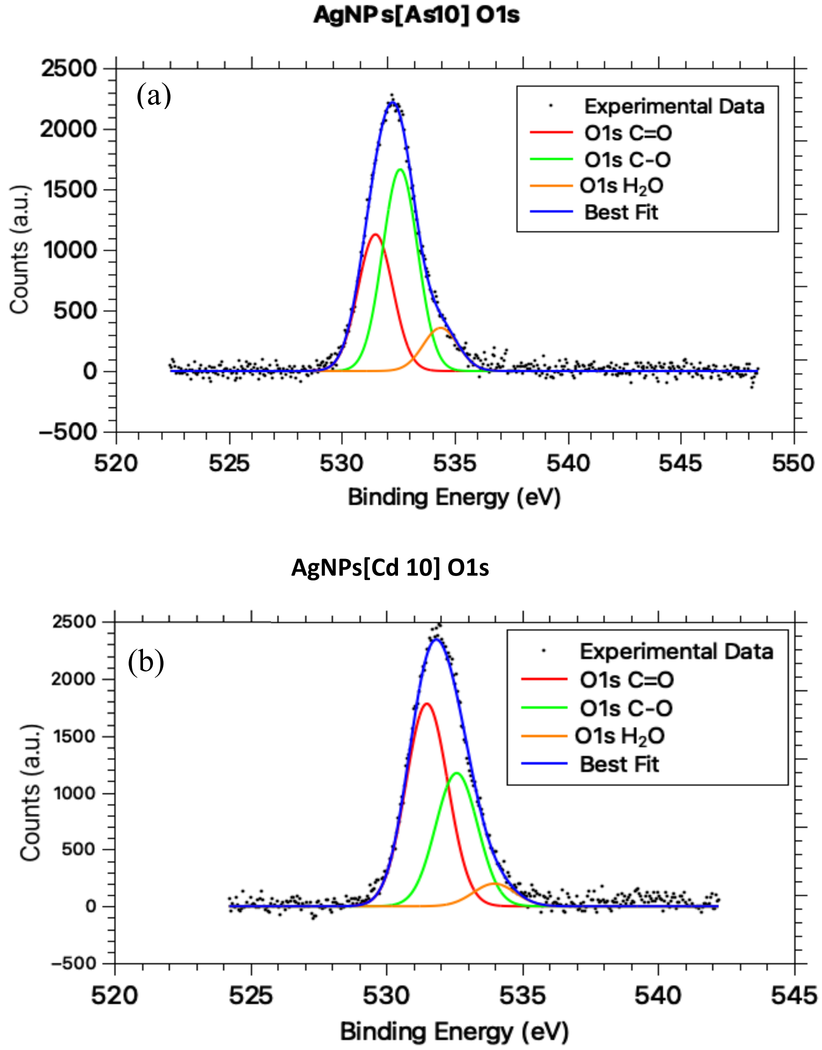
AgNPs[As10] O1s
(a)
Experimental Data
O1s C=O
O1s C-O
O1s H2O
Best Fit
Counts (a.u.)
Binding Energy (eV)
2500
2000
1500
1000
500
0
−500
520
525
530
535
540
545
550
AgNPs[Cd 10] O1s
(b)
Experimental Data
O1s C=O
O1s C-O
O1s H2O
Best Fit
Counts (a.u.)
Binding Energy (eV)
2500
2000
1500
1000
500
0
−500
520
525
530
535
540
545

O1s

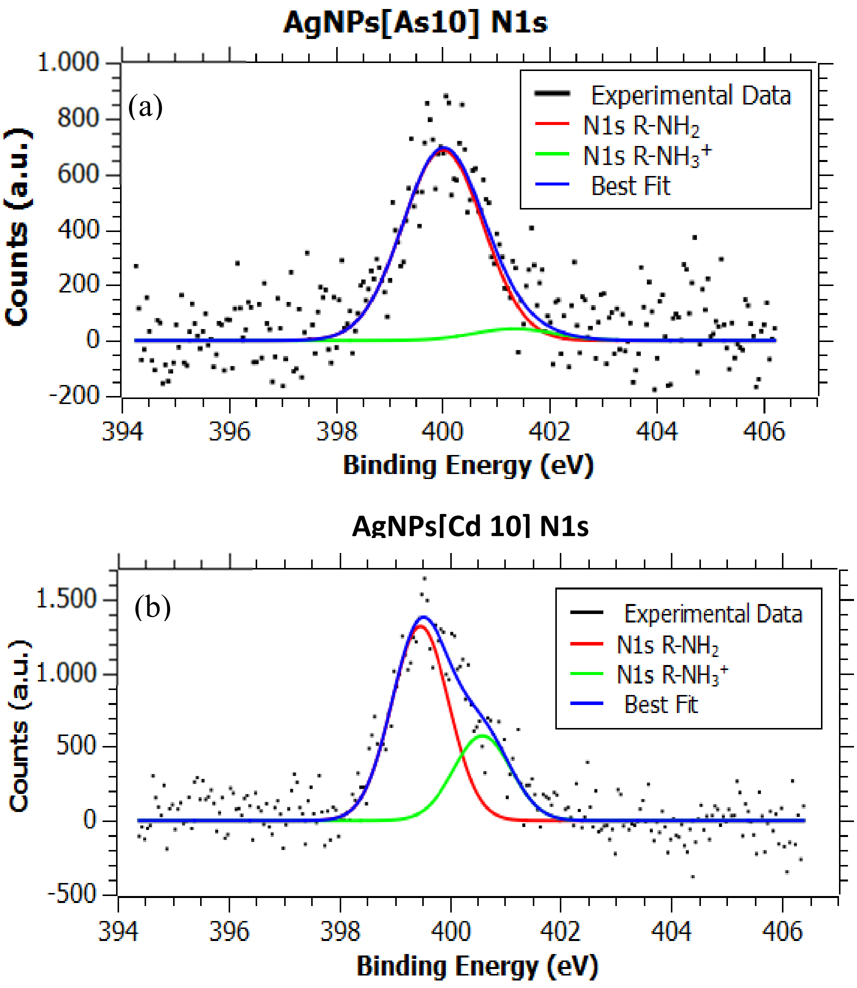

N1s

Figure S2. XPS spectra collected at C1s, O1s and N1s core levels.

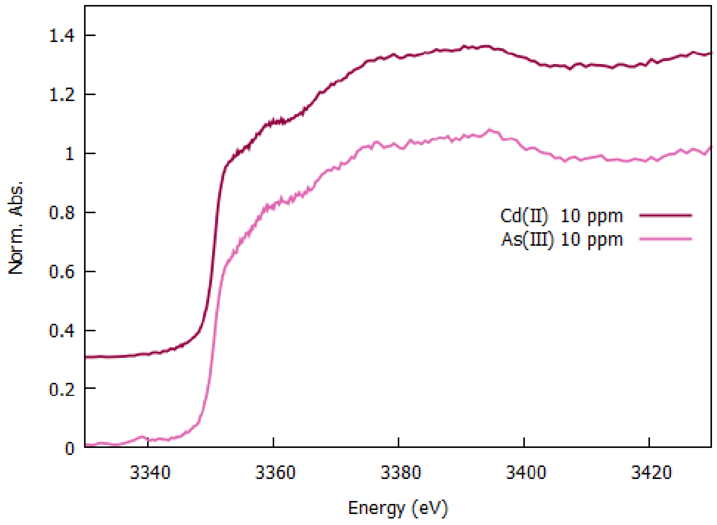

Figure S3. The Ag-L$_3$ edge XANES spectra measured on Ag(III) 10 ppm and Cd(II) 10 ppm samples (vertically shiftd for clarity) confirms the silver mainly in metallic state accordingly to the literature data [1]. The effect of Ag-S coordination at the NP surface is negligible due to the relatively large NP core sizes giving to small surface to volume ratios.

1 Liu, S.H.; Tsai, H.M.; Pao, C.W.; Chiou, J. W.; Ling, D.C.; Pong, W.F.; Tsai, M.-H.; Lin, H.J.; Jang, L.Y.; Lee, J.F.; et al. Electronic and magnetic properties of the Ag-doped Fe 3 O 4 films studied by x-ray absorption spectroscopy. *Appl. Phys. Lett.* **2006**, *89*, 092112.
